# Contrasting Mixotrophic Lifestyles Reveal Different Ecological Niches in Two Closely Related Marine Protists

**DOI:** 10.1111/jpy.12920

**Published:** 2019-11-01

**Authors:** Susanne Wilken, Chang Jae Choi, Alexandra Z. Worden

**Affiliations:** ^1^ Monterey Bay Aquarium Research Institute 7700 Sandholdt Road Moss Landing California 95039 USA; ^2^ Department of Freshwater and Marine Ecology Institute for Biodiversity and Ecosystem Dynamics University of Amsterdam Science Park 904 Amsterdam 1098 XH The Netherlands; ^3^ Ocean EcoSystems Biology Unit GEOMAR Helmholtz Centre for Ocean Research Düsternbrooker Weg 20 Kiel 24105 Germany

**Keywords:** chrysophytes, microbial food web, mixotrophy, phagotrophy, phytoplankton

## Abstract

Many marine microbial eukaryotes combine photosynthetic with phagotrophic nutrition, but incomplete understanding of such mixotrophic protists, their functional diversity, and underlying physiological mechanisms limits the assessment and modeling of their roles in present and future ocean ecosystems. We developed an experimental system to study responses of mixotrophic protists to availability of living prey and light, and used it to characterize contrasting physiological strategies in two stramenopiles in the genus *Ochromonas*. We show that oceanic isolate CCMP1393 is an obligate mixotroph, requiring both light and prey as complementary resources. Interdependence of photosynthesis and heterotrophy in CCMP1393 comprises a significant role of mitochondrial respiration in photosynthetic electron transport. In contrast, coastal isolate CCMP2951 is a facultative mixotroph that can substitute photosynthesis by phagotrophy and hence grow purely heterotrophically in darkness. In contrast to CCMP1393, CCMP2951 also exhibits a marked photoprotection response that integrates non‐photochemical quenching and mitochondrial respiration as electron sink for photosynthetically produced reducing equivalents. Facultative mixotrophs similar to CCMP2951 might be well adapted to variable environments, while obligate mixotrophs similar to CCMP1393 appear capable of resource efficient growth in oligotrophic ocean environments. Thus, the responses of these phylogenetically close protists to the availability of different resources reveals niche differentiation that influences impacts in food webs and leads to opposing carbon cycle roles.

**Abbreviations**
AOX: mitochondrial alternative oxidase
ASW: artificial seawater
C_III_: mitochondrial respiratory complex III
ETR: electron transport rate
FALS: forward‐angle light scatter
FISH: fluorescence in situ hybridization
FRRf: Fast repetition rate fluorometry
GFP: green fluorescent protein
NPQ: non‐photochemical quenching
PTOX: plastid terminal oxidase
VAZ: violaxanthin cycle pigments

Unicellular photosynthetic eukaryotes of diverse evolutionary origin have long been recognized as globally important primary producers in the ocean (Field et al. 1998). As phytoplankton, these pigmented protists are clearly distinguished from purely heterotrophic protists, which are considered the major predators in marine microbial food webs, recycling nutrients, and linking bacterial production to higher trophic levels (Pomeroy 1974, Azam et al. 1983). However, this strict dichotomy does not accurately reflect natural communities, which harbor many mixotrophic protists that combine the ability to photosynthesize with phagotrophic consumption of microbial cells (Bird and Kalff 1986, Estep et al. 1986, Flynn et al. 2013). Mixotrophs can be important consumers of prokaryotic and eukaryotic microbes (Havskum and Hansen 1997, Sanders et al. 2000, Unrein et al. 2007, Hartmann et al. 2012, Orsi et al. 2018), but their concurrent contributions to carbon fixation via photosynthesis complicate integration of their ecosystem roles into biogeochemical models (Mitra et al. 2014, Worden et al. 2015). When both processes are integrated within the same cell, photosynthetic energy acquisition can compensate respirational losses of ingested carbon (Stoecker and Michaels 1991) leading to higher carbon transfer efficiencies from prey to predator, which is predicted to result in more productive food chains (Ward and Follows 2016). Concurrently, higher trophic transfer efficiencies result in lesser release of remineralized nutrients by mixotrophic compared to heterotrophic predators (Rothhaupt 1997). However, the net impact of phagotrophic mixotrophs on carbon cycling may vary widely between species or environmental conditions, creating considerable uncertainty in how these organisms should be included in ecosystem models. The accuracy of theory‐based predictions that involve mixotrophy, such as trait‐based approaches (Andersen et al. 2015) or detailed physiological models (Flynn and Mitra 2009), is currently limited by the weak empirical basis for model assumptions and parameterization.

The combined capacity for photosynthesis and phagocytosis in many extant eukaryotes is not surprising. Phagocytosis is an ancient trait that has been involved in acquiring photosynthetic cells as endosymbionts, giving rise to the first eukaryotic photosynthetic organelle, the plastid, and then spreading into evolutionarily distant lineages of eukaryotes through additional endosymbiosis events (Lane and Archibald 2008, Keeling et al. 2013, Worden et al. 2015). In some photosynthetic eukaryotes, the capacity for phagocytosis has been lost, such as diatoms, which belong to the stramenopiles. However, the presence of mixotrophs across distant branches of the eukaryotic tree (such as multiple independent lineages within the alveolates, prymnesiophytes, and stramenopiles), with different evolutionary histories, cellular morphologies, and metabolic potentials, suggests that plastid acquisition does not necessarily result in evolution toward specialist photoautotrophy (Selosse et al. 2017). Despite the phylogenetic diversity among mixotrophs their lifestyles can be classified functionally into non‐constitutive mixotrophs that acquire their photosynthetic potential by hosting photosynthetic endosymbionts or stealing plastids, and constitutive mixotrophs that possess their own plastids (Mitra et al. 2016). Additionally, mixotrophic strategies vary in the degree to which photoautotrophy and phago‐heterotrophy contribute to the overall nutrition (Stoecker 1998) and the phenotypic plasticity that allows adjusting the strategy in response to environmental conditions. Recent attempts have started to address this functional diversity of mixotrophs in models (Flynn and Mitra 2009, Mitra et al. 2016, Berge et al. 2017, Leles et al. 2018), but underlying metabolic attributes will need to be resolved to understand the biogeochemical and ecological impact as well as evolutionary trajectories of photosynthetic eukaryotes.

The taxonomic identity of the plastid‐containing constitutive mixotrophs that often dominate bacterivory in marine environments is not well characterized and studies quantifying their impact rarely report community composition (but see Unrein et al. 2014). Multiple prymnesiophytes and a specific group within the stramenopiles, the chrysophytes, have been identified as mixotrophic bacterivores in oligotrophic ocean regions (Hartmann et al. 2013). The few studies that have evaluated chrysophyte abundances, either by enumeration of plastid‐containing chrysophytes using FISH (Jardillier et al. 2010) or by semi‐quantitative dot‐blot hybridization (Lepère et al. 2009, Kirkham et al. 2013), identified them as a numerically important component of pico‐ and nanophytoplankton communities in the tropical North‐East Atlantic, the hyper‐oligotrophic South Pacific gyre, the Arctic and Indian oceans. Chrysophyte sequences retrieved from environmental assays are typically dominated by environmental clades (Lepère et al. 2009, del Campo and Massana 2011), and the roles of individual clades in the marine microbial food web remain unknown. Chrysophytes include species with numerous nutritional strategies from autotrophic to various mixotrophic and purely heterotrophic lifestyles, with loss of photosynthetic capabilities having occurred multiple times independently (Boenigk et al. 2005, Grossmann et al. 2016). However, physiological information for this group is largely based on cultured isolates from freshwater (Caron et al. 1990, Rothhaupt 1996), brackish, and coastal environments (Andersson et al. 1989, Flöder et al. 2006). It is unclear how far information from coastal isolates or other evolutionarily distant groups such as dinoflagellates can be extrapolated to oceanic chrysophytes. The sole study of an oceanic chrysophyte, *Ochromonas* CCMP1393, uses transcriptome analyses to infer that it has a stronger dependence on photosynthesis than a freshwater isolate (Lie et al. 2018). Mixotrophic strategies observed in freshwater and brackish *Ochromonas* species often show a strong heterotrophic component with the ability to grow purely heterotrophically in darkness (Tittel et al. 2003, Pålsson and Daniel 2004). A close interaction between photosynthetic and mitochondrial electron flow as also known from diatoms (Allen et al. 2008, Bailleul et al. 2015) has been hypothesized to underlie a tendency toward photoheterotrophy in the freshwater *O. danica* (Wilken et al. 2014a). However, *Ochromona*s as defined today appears to be polyphyletic (Grossmann et al. 2016) and the physiology of marine isolates has yet to be directly compared in feeding experiments.

The nutritional diversity of chrysophytes provides a platform for investigating differentiated strategies in phylogenetically related organisms from various environments. The challenges to studying energetics and implications of different mixotrophic strategies lie principally in the maintenance of predatory mixotrophs in culture under axenic conditions and performing experiments with living prey. While mesocosm studies have employed bacteria expressing green fluorescent protein (GFP) as experimental amendments of traceable living prey (Worden et al. 2006), this approach has yet to be employed in culture‐based studies. Here we developed a controlled experimental system to manipulate and track the availability of live bacterial prey, using a marine *Vibrio* mutant that has a higher temperature optimum (Graf et al. 1994) than the eukaryotes under study and harbors GFP on an antibiotic resistance plasmid. These systems were developed for two marine chrysophytes expected to represent different species, the coastal (CCMP2951) and oceanic (CCMP1393) *Ochromonas* isolates. We compared the relative importance of photosynthesis and ingestion of bacterial prey to nutrition of both isolates and assessed the phenotypic plasticity in response to variations in light intensity and prey availability. To examine differences in their lifestyles more deeply, we further tested for a potential interaction between nutritional pathways, by assessing the role of mitochondrial respiration in photosynthetic electron transport. Our study provides first insights into the underlying metabolic and physiological characteristics of distinct nutritional strategies in predatory mixotrophs from both coastal and oceanic environments. Collectively, such studies will improve our ability to mechanistically integrate widespread mixotrophic lifestyles into models used to predict carbon cycling under future ocean conditions.

## Materials and Methods

### Chrysophyte isolates and growth conditions

The two *Ochromonas* isolates, CCMP1393 and CCMP2951, were obtained from the National Center for Marine Algae and Microbiota (NCMA, East Boothbay, ME, USA). They originate from the North‐West Atlantic Gulf Stream (38.70° N, 72.37° W) and a coastal site in the subtropical North‐West Pacific (24.76° N, 125.44° E), respectively. Morphological differences indicate that they might represent different species with an eyespot being present in CCMP2951 (as documented on the NCMA website: https://ncma.bigelow.org/ccmp2951#.XNvQzcTgpPY) but not CCMP1393. The cultures were grown on K‐medium containing both ammonium (50 μM) and nitrate (882 μM) as nitrogen source and Na_2_‐glycerophosphate (10 μM) as phosphorus source (Keller et al. 1987) in an artificial seawater (ASW) base with a salinity of 34. This medium was chosen because it had been developed specifically for oceanic microalgae and because the presence of ammonium and glycerophosphate would prevent impaired photosynthetic growth of *Ochromonas* due to potential inaccessibility of standard inorganic nutrient sources such as nitrate (Wilken et al. 2014b). However, we note the possibility of the organic phosphorus source to also support low levels of osmotrophy. Cultures received additions of heat‐killed bacterial prey (see below) and were maintained at 21°C on a 14:10 h light:dark cycle with a light intensity of 100 μmol photons · m^−2^ · s^−1^ unless indicated otherwise.

### Development of controlled prey experimental system

The two chrysophytes were rendered axenic by killing their undefined bacterial consortia using antibiotics. Specifically, they were incubated with 8 μg · mL^−1^ chloramphenicol for 24 h, transferred into medium containing 40 μg · mL^−1^ kanamycin, 80 μg · mL^−1^ neomycin, 100 μg · mL^−1^ streptomycin, and 150 μg · mL^−1^ penicillin G, and grown in this antibiotic cocktail with transfer and feeding with heat‐killed bacterial prey (see below) every other day for a total of 2 weeks. Success of the antibiotic treatment was verified by testing for bacterial growth in a medium containing peptone and malt extract, and by epifluorescence microscopy based on staining with 15 nM 4′,6‐Diamidine‐2′‐phenylindole dihydrochloride (DAPI).

The non‐motile FlrA‐mutant of *Vibrio fischeri* ES114 (Millikan and Ruby 2003) was used as both a heat‐killed bacterial prey source and a traceable living food source. Introduction of the pVSV102 plasmid further conferred kanamycin resistance and constitutive expression of GFP (Dunn et al. 2006). Stock cultures of the mutant were maintained on LBS plates (Stabb et al. 2001) with 100 μg · mL^−1^ kanamycin at room temperature. Prior to experiments, *V. fischeri* was transferred into liquid medium containing 100 μM phosphate, 821 μM ammonium chloride, 888 μM glucose, K‐medium trace metal mix (Keller et al. 1987), 4 mM Tris HCl pH 7.5, and 100 μg · mL^−1^ kanamycin in an ASW base. Bacteria were harvested in exponential phase (abundance between 0.8 and 1.3 × 10^9^ cells · mL^−1^) and either heat‐killed at 80°C for 30 min (for maintenance of chrysophyte stock cultures) or used as live bacterial prey for experiments. Bacterial cells were centrifuged at 10,000*g* for 12 min, washed in KASW medium, and stored at 4°C overnight prior to use in experiments.

### Flow cytometry

Cells were enumerated by flow cytometry after fixation with freshly prepared, buffered formaldehyde (pH 7.2; 1% final concentration) and dark incubation for 20 min. Fixation in this manner did not induce visible prey egestion. Fixed samples were either run immediately on an Accuri C6 flow cytometer (BD Biosciences, San Jose, CA, USA) or flash‐frozen in liquid nitrogen and stored at −80°C until analysis on an Influx flow cytometer (BD Biosciences; for details see: Cuvelier et al. 2010). Red and yellow‐green polystyrene fluorescent beads (Polysciences, Warrington, PA, USA) were added as internal standards. On the Accuri, the detection threshold was set on forward‐angle light scatter (FALS) for *Ochromonas* and on green fluorescence (510/15 nm) for *V. fischeri*. For the latter, settings were validated against flow‐cytometric bacterial counts after DNA staining with SybrGreen (Invitrogen), to ensure that the GFP signal was sufficient to detect all bacteria. The chlorophyll‐derived autofluorescence of *Ochromonas* cells was normalized to the red beads and represents a proxy for chlorophyll content (Cuvelier et al. 2017).

### DNA extraction, sequencing, and phylogenetic analyses

CCMP2951 cells were harvested from 50 mL culture by centrifugation at 8,000*g* for 10 min. DNA was extracted using the DNeasy Plant Mini Kit (Qiagen, Valencia, CA, USA). Polymerase chain reaction (PCR) to amplify the 18S rRNA gene was performed using forward primer 5′‐ACCTGGTTGATCCTGCCAG‐3′ and reverse primer 5′‐TGATCCTTCYGCAGGTTCAC‐3′ (Moon‐van der Staay et al. 2000) in a total volume of 25 μL containing 0.2 μM of each primer, 1 μL template, and 12.5 μL of HotStarTaq MasterMix (Qiagen). After initial activation at 95°C for 15 min, 30 amplification cycles were performed consisting of 30 s at 95°C, 30 s at 50°C, 4 min at 72°C, followed by 10 min final extension at 72°C. PCR products were purified by electrophoresis, gel extracted (QIAquick Gel Extraction Kit; Qiagen), and cloned with the TOPO‐TA cloning kit (Invitrogen, Carlsbad, CA, USA). Plasmids were Sanger‐sequenced bi‐directionally using plasmid primers M13F/M13R and the two internal primers 502f and 1174r (Worden 2006). The resulting 1,786 bp CCMP2951 18S rRNA gene sequence was deposited under accession MH420530 (NCBI NR).

Near full‐length 18S rRNA gene sequences representing chrysophyte diversity were retrieved from the SILVA database (release 128, https://www.arb-silva.de/documentation/release-128/; Pruesse et al. 2007). These 126 sequences, 4 additional sequences retrieved from iMicrobe MMETSP database (Keeling et al. 2014), alongside the CCMP2951 sequence generated herein and a Synchromophyceae sequence (as outgroup) were aligned using MAFFT (Katoh and Standley 2013) with default parameters. Gaps were masked using Gblocks (Castresana 2000) and phylogenetic inferences made by maximum likelihood methods based on 1,444 homologous positions implemented in RAxML (Stamatakis 2014) under the gamma corrected GTR model of evolution with 1,000 bootstrap replicates, or PhyML 3.0.1 (Guindon et al. 2010) with the same substitution model and 100 bootstrap replicates. Additional reconstructions were performed in MrBayes 3.2.6 (Ronquist et al. 2012) and the final tree was produced using FigTree 1.4.0 (http://tree.bio.ed.ac.uk/software/figtree) and RAxML topology. Literature searches were performed for all genera represented in the tree to retrieve information on their capabilities to perform photosynthesis and phagocytosis. We note that detection of phagotrophic capacity requires a targeted experiment while photosynthetic capacity is inferred from readily detectable pigmentation of cells.

### Experiments

Prior to experiments, the *Ochromonas* isolates were acclimated to experimental conditions by maintenance in semi‐continuous culture for 10 generations with both light and prey being available. To achieve this, cell abundances were kept between 5 and 10 × 10^4^ cells · mL^−1^ and live bacterial prey was added daily to an abundance of 3 × 10^7^ cells · mL^−1^. These abundances were established in preliminary feeding experiments to avoid prey depletion to sub‐saturating abundances below 1 × 10^7^ cells · mL^−1^ within 24 h (Rothhaupt 1996), but allow depletion of prey by three orders of magnitude over a longer period (3 d). Daily dilution of cultures with fresh medium and supplementation of live bacterial prey was based on flow cytometry counts of both predator and prey.

Three experiments were performed using the GFP traceable, live bacterial prey. Experiment 1 addressed the growth response to light intensity under mixotrophic conditions (with prey‐amendment), Experiment 2 addressed the growth response to availability of light and prey, and Experiment 3 assessed physiological responses to light limitation and prey depletion.

For Experiment 1, light–response curves were performed in triplicate for both isolates under prey‐amended conditions. The light intensities used examined the same range of light levels, with slight differences in specific levels between isolate experiments, with CCMP1393 grown at 0, 15, 30, 80, 130, and 210 μmol photons · m^−2^ · s^−1^ and CCMP2951 at 0, 20, 40, 57, 95, and 165 μmol photons · m^−2^ · s^−1^. Cells were enumerated daily by flow cytometry (Accuri C6) as described above. Biological triplicates of each isolate were maintained for 10 generations and specific growth rates were then averaged over the last 5 d of the growth period for each biological replicate.

Experiment 2 further assessed the potential for purely autotrophic or heterotrophic growth. To this end, triplicate cultures of both isolates were pre‐grown mixotrophically with daily prey‐amendments (as described above) at a light intensity of 100 μmol photons · m^−2^ · s^−1^, close to the optimum determined for each isolate (see results). Cultures were then split into (i) an autotrophic treatment that did not receive additional prey; (ii) a mixotrophic treatment that continued to receive daily prey‐amendments; and (iii) a heterotrophic treatment that received daily prey‐amendments, but was kept in darkness. Cultures were maintained for 7 d as described above, sampled daily, and enumerated by flow cytometry.

For Experiment 3, triplicate cultures of both isolates were acclimated to limiting (low) light intensity of 15 or near‐optimal (high) light intensity of 100 μmol photons · m^−2^ · s^−1^, respectively, and maintained for 10 generations as described above. Cultures were then split into a mixotrophic, prey‐amended treatment grown with continued addition of bacterial prey as before and a prey‐deplete treatment that did not receive additional prey. Both treatments were grown semi‐continuously for four more days with daily sampling for flow cytometry and dilution to 5 × 10^4^ cells · mL^−1^. Photo‐physiological sampling and measurements were performed after 3 d, when both isolates still exhibited positive growth rates when receiving sufficient light (i.e., 100 μmol photons · m^−2^ · s^−1^), while afterwards CCMP2951 ceased growing in prey‐deplete treatments. This experiment thus assessed the short‐term response to prey depletion rather than acclimated autotrophic growth, which the two isolates were not capable of. Samples were taken for pigment analysis and measurements of both carbon fixation and prey ingestion rates. Fast repetition rate fluorometry (FRRf) was used to record rapid light–response curves and the response of photosynthetic electron transport to chemical inhibition of mitochondrial respiration or chlororespiration.

#### Pigment analysis

For pigment analysis, 50 mL of culture was filtered onto 25‐mm GF/F filters (Whatman, GE Healthcare Life Sciences, Pittsburgh, PA, USA), frozen in liquid N_2_, and stored at −80°C. Extraction and HPLC analysis were performed at the analytical facility of Horn Point Laboratory at the University of Maryland Center for Environmental Science. Concentrations of chlorophyll a (Chl*a*), fucoxanthin, carotene, diadinoxanthin and the violaxanthin cycle pigments (VAZ) violaxanthin (V), antheraxanthin (A), and zeaxanthin (Z) were quantified according to Van Heukelem and Thomas (2001).

#### Carbon fixation

Net rates of carbon fixation were measured as ^14^C‐incorporation over 24 h. This incubation period was chosen because it reliably provides net rates of carbon fixation, while shorter‐term incubations can result in estimates somewhere between gross and net rates, depending on species and growth conditions (Milligan et al. 2015). For each of the triplicate cultures two 20 mL aliquots at 5 × 10^4^ cells · mL^−1^ of *Ochromonas* received 250 μCi ^14^C‐bicarbonate at the onset of the light period. Depending on the treatment, the cultures contained either 3 × 10^7^ cells mL^−1^ of *Vibrio fischeri* (prey‐amended treatments) or no prey (prey‐deplete treatments). One aliquot was incubated with light intensity and diel cycle matching acclimation conditions (15 or 100 μmol photons · m^−2^ · s^−1^) and the second served as dark control. After 24 h incubation, 100 μL samples from ^14^C‐incubations was added to 10 mL scintillation cocktail (CytoScint; MP Biomedicals, Santa Ana, CA, USA) to measure the total ^14^C‐activity. The remaining cultures from ^14^C‐incubations were filtered onto 25‐mm GF/F filters to measure activities in samples and dark control, respectively. Filters were incubated in 5% HCl overnight to remove inorganic ^14^C. After receiving 10 mL of scintillation, cocktail samples were measured on a scintillation counter (Beckman Coulter LS 6500, Indianapolis, IN, USA). Rates of carbon fixation were calculated according to Pennington and Chavez (2000) and converted to cellular rates of carbon fixation using the mean *Ochromonas* abundance accounting for growth over the incubation time (Heinbokel 1978).

#### Ingestion rates

Ingestion rates were measured by prey disappearance relative to a prey only control. For each strain and light intensity, triplicate cultures were diluted to 5 × 10^4^ cells mL^−1^ and *Vibrio fischeri* was added to a final abundance of 3 × 10^7^ cells · mL^−1^. Triplicate control flasks received the same abundance of *V. fischeri* and a volume of culture filtrate (0.2 μm) equal to the culture volume added to the grazing treatment. Flow cytometry samples were taken after 0 and 24 h of incubation and fixed, stored, and counted on the Influx as described above. Ingestion rates were calculated according to Heinbokel (1978). For measurement of prey carbon content, duplicate 20 mL aliquots of *V. fischeri* stock used as bacterial prey were filtered onto pre‐combusted GF‐75 filters (Advantec, Dublin, CA, USA), stored at −20°C and dried at 50°C overnight prior to analysis at the analytical facility of Horn Point Laboratory at the University of Maryland, Center for Environmental Science. Live *V. fischeri* used for the experiments had a carbon content of 0.069 ± 0.014 pg C · cell^−1^.

#### Fluorescence kinetics of photosystem II (PSII)

FRRf was used to measure PSII fluorescence kinetics. Rapid light–response curves were recorded using a sequence of eight light intensities from 0 to 300 μmol photons · m^−2^ · s^−1^. Cultures were exposed to the respective light intensity for 3 min before performing four series of measurements at 20 s time intervals. For dark acclimated readings (*F*_0_ and *F*_m_), cultures were incubated in darkness for 25 min prior to measurements. The excitation and relaxation protocols are described in Guo et al. (2018). During excitation, fluorescence rises from a minimum (*F*_0_) or steady‐state (*F* ′_s_) value in dark‐acclimated cultures or in actinic light, respectively, to a maximum (*F*_m_ or *F* ′_m_ in darkness or actinic light, respectively) as reaction centers close during light saturation. Fluorescence parameters and the effective absorption cross‐section of PSII (σ_PSII_ in Å^2^) were derived according to Kolber et al. (1998). Effective quantum efficiency of PSII was derived as Δ*F*/*F*′_m_=(*F*′_m_ − *F*′_s_)/*F*′_m_ and maximum quantum efficiencies were calculated from dark acclimated readings as *F*_v_/*F*_m_=(*F*_m_ − *F*_0_)/*F*_m_. Rates of electron transport per functional reaction center of PSII (ETR_RCII_) were estimated as in Schuback et al. (2015):

$${\text{ETR}}_{\mathrm{RCII}}=E\times \sigma_{\mathrm{PSII}}\times \left(\frac{\Delta F/{F^{\prime }}_{m}}{F_{v}/F_{m}}\right)\times 6.022\times 10^{-3}$$

where *E* is irradiance and the factor 6.022 × 10^−3^ converts μmol photons to photons and Å^2^ to m^2^. The exponential photosynthesis–irradiance curve (Webb et al. 1974)

$$\text{ETR}={\text{ETR}}_{\max}\left(1-e^{-\frac{\alpha E}{{\mathrm{ETR}}_{\max}}}\right)$$

was fitted to ETR_RCII_ data from rapid light–response curves using the iterative least‐square nonlinear regression in Sigma‐Plot 13. Non‐photochemical quenching (NPQ) was calculated as:

$$\text{NPQ}=\frac{\left(F_{m}-{F^{\prime }}_{m}\right)}{{F^{\prime }}_{m}}.$$

CCMP1393 exhibited maximum fluorescence values (*F*_max_) in low light instead of darkness and *F*_max_ therefore replaced *F*_m_ in the calculation of NPQ.

#### Inhibition of mitochondrial and chloro‐respiration

The influence of mitochondrial and chloro‐respiration on electron transport through PSII was assessed by FRRf measurements in the presence of inhibitors. Plastid terminal oxidase (PTOX), the electron acceptor in chlororespiration, was inhibited by 1 mM propylgallate (Bailey et al. 2008). The mitochondrial alternative oxidase (AOX) and respiratory complex III (C_III_) were inhibited by 1 mM salicylhydroxamic acid and 5 μM antimycin A, respectively (Bailleul et al. 2015). A combination of salicylhydroxamic acid and antimycin A was used to block mitochondrial respiration completely. Readings were taken using prey‐amended cultures pre‐acclimated to 100 μmol photons · m^−2^ · s^−1^ and subjected to 3 min of illumination by 20, 100, and 300 μmol photons · m^−2^ · s^−1^ prior to the measurement representative of sub‐saturating, near‐saturating, and super‐saturating light intensities relative to acclimation conditions. The effect of mitochondrial inhibitors on photosynthetic electron transport is expressed as relative decrease in the effective quantum efficiency of PSII (Δ*F*/*F* ′_m_) compared to the control reading acquired before addition of inhibitors.

#### Statistics

To detect differences in growth between autotrophic, mixotrophic, and heterotrophic conditions over time growth curves were analyzed by repeated measures ANOVA. Pigment contents and carbon fixation were tested for differences between isolates as well as effects of light intensity and prey availability by three‐way ANOVA. Effects of light and prey availability were also analyzed separately for each isolate by two‐way ANOVA to assess their impact and interaction in each isolate individually. Because ingestion rates can only be measured in prey‐amended treatments, a two‐way ANOVA was used for the full data set, while a one‐way ANOVA tested for the effect of light in each isolate individually. Carotene to Chl*a* ratios were log‐transformed to improve homoscedasticity. FRRf measurements of rapid light–response curves and inhibitor tests were analyzed by repeated measures ANOVA with light as repeated factor. Holm‐Sidak tests were performed for all post‐hoc pairwise comparisons.

## Results

### Phylogenetic tree of chrysophytes

**Figure 1:**
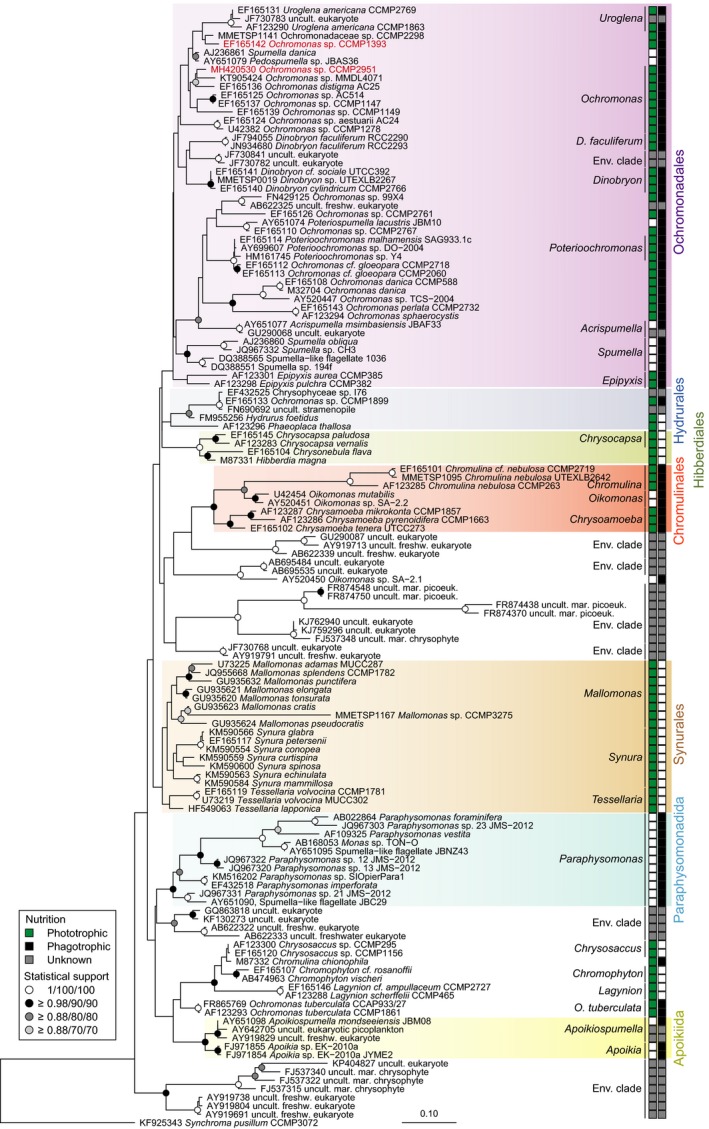
Phylogenetic reconstruction of chrysophytes based on 18S rRNA gene sequences. A total of 1,444 positions from 132 near full‐length sequences were used. Tree topology is based on maximum likelihood inference and node statistical supports are indicated based on Bayesian posterior node probabilities, and percent of bootstrap replicates from two maximum likelihood methods (1,000 replicates in RAxML and 100 replicates in PhyML). Columns of colored squares indicate the nutritional capacities of described genera for photosynthesis (left column: green—known phototrophic potential, white—potential not reported in cultured isolates, grey—unknown potential) and for phagotrophic ingestion of prey (right column: black—known phagotrophic potential, white—potential not reported in cultured isolates, grey—unknown potential). [Color figure can be viewed at http://www.wileyonlinelibrary.com]

The distribution of nutritional strategies across the phylogenetic tree of chrysophytes is complex, based on the current level of understanding and available isolates (Fig. 1). The orders Hibberdiales and Synurales are composed of presumably purely photosynthetic organisms, while Paraphysomonadida and Apoikiida contain solely non‐photosynthetic organisms. The two isolates studied here have 97% nucleotide identity between their 18S rRNA genes and belong to the Ochromonadales, a group which contains both mixotrophic and purely heterotrophic taxa. Moreover, the presence of heterotrophic taxa in several Ochromonadales subclades suggests photosynthetic machinery has been lost multiple times within this group.

### Light–response curves (Experiment 1)

**Figure 2:**
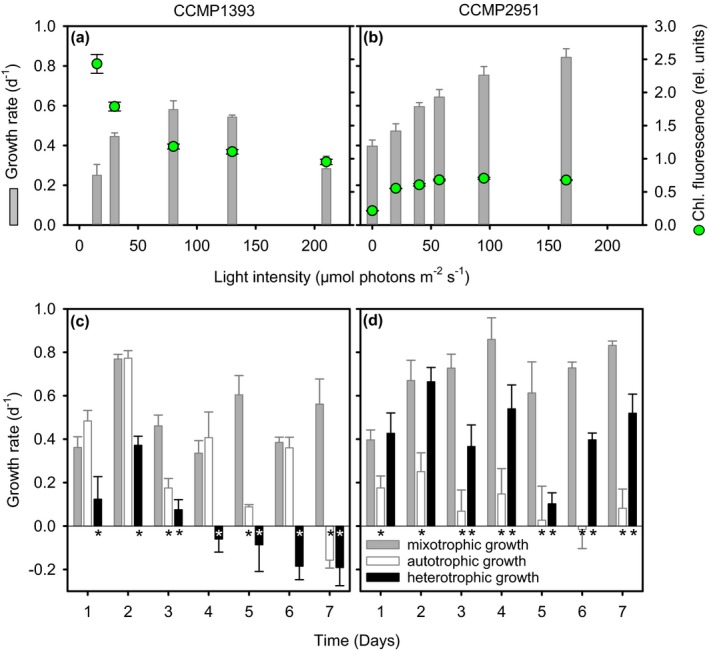
Growth responses to light under prey‐amended conditions in the two *Ochromonas* isolates. (a and b) Light–response curves and cellular chlorophyll *a* fluorescence of CCMP1393 and CCMP2951 during prey‐amended growth in the light (mixotrophic growth; Experiment 1). Cultures were maintained semi‐continuously with daily additions of *Vibrio fischeri* as prey. (c and d) Growth curves of CCMP1393 and CCMP2951 (Experiment 2) with daily prey‐amendments in the light (mixotrophic growth), with daily prey‐amendments in darkness (heterotrophic growth), or after ceasing prey‐amendments in the light (autotrophic growth). Asterisk indicates significant difference from mixotrophic conditions (*P *< 0.05; Holm‐Sidak test following a two‐way repeated measures ANOVA). Error bars indicate standard deviation among biological triplicates. [Color figure can be viewed at http://www.wileyonlinelibrary.com]

The two isolates of *Ochromonas* showed pronounced differences in their responses to resource availability. When both light and prey were available to support mixotrophic nutrition CCMP1393 and CCMP2951 reached maximum growth rates of 0.58 ± 0.04 and 0.82 ± 0.04 · d^−1^, respectively (Fig. 2, a and b). Under these conditions, differences in responses to light intensity were reflected in adjustments of cellular red fluorescence levels. CCMP1393 showed increased red fluorescence at low‐light intensities (Fig. 2a; ANOVA: *F*_4,10_ = 196.8, *P *< 0.001), while CCMP2951 showed decreased fluorescence when shifting to a more heterotrophic nutrition at low‐light intensities (Fig. 2b; ANOVA: *F*_5,12_ = 825.7, *P *< 0.001). CCMP1393 exhibited photoinhibition at 210 μmol photons · m^−2^ · s^−1^, while CCMP2951 did not show signs of photoinhibition over the range of light levels tested (maximum 180 μmol photons m^−2^ s^−1^).

### Autotrophic and heterotrophic growth potential (Experiment 2)

When kept in darkness, CCMP1393 started to die after 3 d (Fig. 2c), while CCMP2951 could maintain growth rates of 0.40 ± 0.03 · d^−1^ by heterotrophy with bacterial prey as the only source of carbon. After daily prey‐amendments had ceased, CCMP1393 maintained relatively high growth rates for a few days after depleting prey, whereas CCMP2951 showed an immediate drop in growth rates (Fig. 2, c and d). However, after 7 d without feeding CCMP1393 began to die while CCMP2951 did not exhibit measureable death rates.

### Response to light limitation and depletion of prey (Experiment 3)

**Figure 3:**
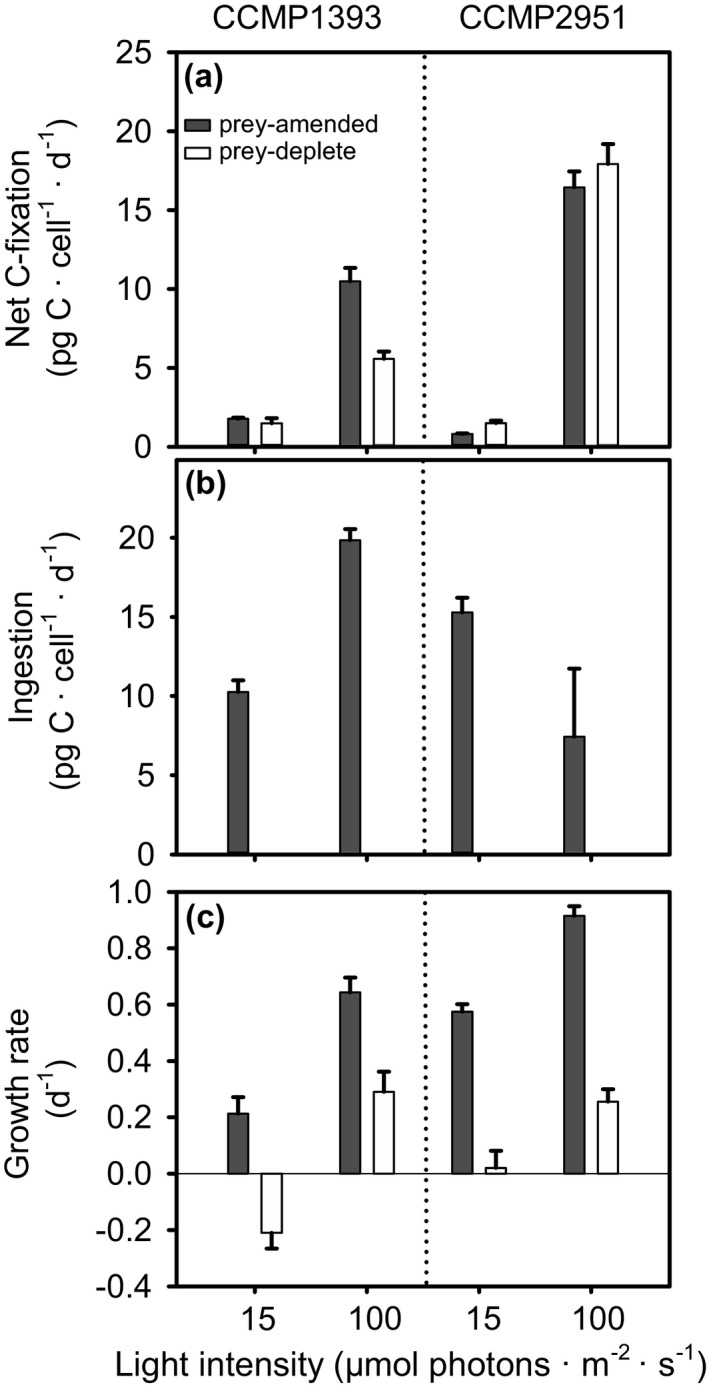
Carbon acquisition and growth of the two *Ochromonas* isolates under prey‐amended and deplete conditions. Rates of (a) net primary production, (b) ingestion, and (c) growth in CCMP1393 and CCMP2951 acclimated to 15 (LL) or 100 (HL) μmol photons · m^−2^ · s^−1^ under either prey‐amended or prey‐deplete conditions. Error bars indicate standard deviation among biological triplicates. For figure simplicity, results of statistical comparisons are shown in Table 1.

**Figure 4:**
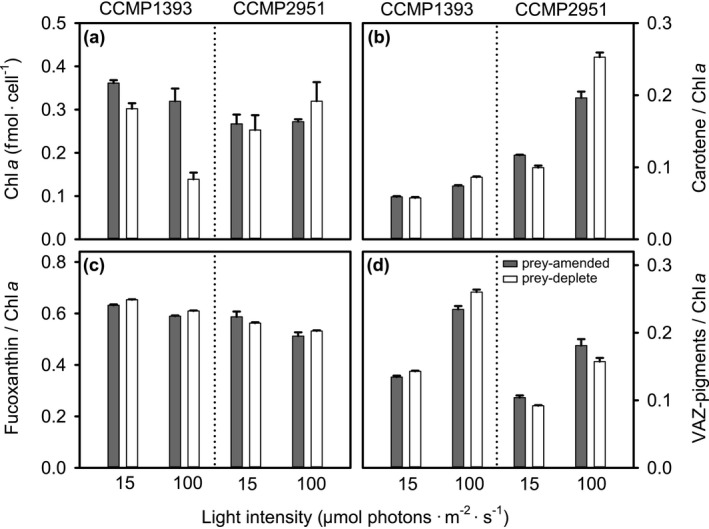
Pigmentation of the two *Ochromonas* isolates under prey‐amended and prey‐deplete conditions. (a) Cellular chlorophyll content and relative content of (b) carotene, (c) fucoxanthin, and (c) Violaxanthin cycle pigments (VAZ) in CCMP1393 and CCMP2951 acclimated to 15 (LL) or 100 (HL) μmol photons · m^−2^ · s^−1^ under either prey‐amended or prey‐deplete conditions. Relative pigment contents are expressed as molar ratios to Chl *a*. Error bars indicate standard deviation among biological triplicates. For figure simplicity, results of statistical comparisons are shown in Table 1.

In line with these different resource requirements, the two isolates responded differently to light limitation and the depletion of prey over a period of 3 d. Time courses of prey depletion were similar among isolates, although CCMP2951 depleted its prey less strongly at low‐light intensities (Figure S1 in the Supporting Information). In CCMP1393, photosynthetic carbon fixation was nearly halved after prey had been depleted in the high‐light treatment, while it remained unchanged in CCMP2951 (Fig. 3a; two‐way ANOVA, Table 1). For both isolates, the 7‐fold decrease in light intensity, from near‐optimal to limiting conditions, significantly reduced net rates of carbon fixation (Fig. 3a; three‐way ANOVA, effect of light, Table 1), although their responses differed significantly (three‐way ANOVA, light × isolate interaction, Table 1). While rates of carbon fixation decreased 12‐ to 20‐fold in CCMP2951, they only decreased 4‐ to 6‐fold in CCMP1393 (Fig. 3a). This difference is in line with the increased chlorophyll content of CCMP1393 at low‐light intensities (Fig. 4a; two‐way ANOVA, Table 1). Ingestion rates showed a different pattern to cellular chlorophyll content with a significant increase at higher light intensity in CCMP1393 and decrease in CCMP2951 (Fig. 3b; two‐way ANOVAs, Table 1). Hence, in prey‐amended cultures carbon fixation and ingestion covaried in response to light intensity in CCMP1393, while they were inversely related in CCMP2951. Although low prey and light availabilities both led to reduced growth in both isolates (Fig. 3c; *P *< 0.001, Table 1), their responses to these resources differed significantly (two‐way ANOVA interaction terms, Table 1). CCMP1393 grew generally faster in high‐light conditions, while CCMP2951 always showed higher growth rates when prey‐amended. Overall, the larger dependency on photosynthesis in CCMP1393 resulted in a relatively stronger reduction of growth under low‐light conditions (67%) compared to CCMP2951 (37%). Under low‐light carbon fixation was not sufficient for survival of CCMP1393 after prey depletion resulting in a population decrease, while at light‐saturation the decrease in growth after prey depletion (55%) was less severe than in CCMP2951 (72%). Note that ingestion rates represent gross carbon uptake from prey, while carbon fixation is represented by net rates. Therefore, measured rates of carbon acquisition cannot be directly linked to growth responses. A decrease in FALS upon prey depletion (Figure S2 in the Supporting Information) indicates decreased cell sizes, which might further skew the relationship between cellular carbon acquisition and specific growth rates.

**Table 1:** Statistical analysis of results from Experiment 3. Effects of *Ochromonas* isolate phylogenetic identity, light intensity, and prey availability as well as their interactions in a three‐way ANOVA and effects of light intensity, and prey availability as well as their interaction in each isolate individually by a two‐way ANOVA. Shown are effects on carbon fixation (Cfix), rates of ingestion and growth, as well as Chl*a* content, and relative contents of carotene (Caro), fucoxanthin (Fuco), and violaxanthin cycle pigments (VAZ). Arrows indicate the direction of significant responses with increasing light intensity or prey availability, respectively

Parameter	Factor	df1	df2	Three‐way ANOVA				Two‐way ANOVA	
				Single effect		Isolate Interaction		CCMP1393	CCMP2951
				*F*	*P*	*F*	*P*		
Cfix/ cell	Isolate	1	16	26.6	<0.001				
Cfix/ cell	Light	1	16	2668	<0.001	204.2	<0.001	↑^a^	↑^a^
Cfix/ cell	Prey	1	16	0.64	0.436	83.0	<0.001	↑^a^	‐a
Ingestion	Isolate	1	8	8.01	0.022				
Ingestion	Light	1	8	0.44	0.526	44.8	<0.001	↑	↓^a^
Growth	Isolate	1	16	93.9	<0.001				
Growth	Light	1	16	309.9	<0.001	17.2	0.001	↑^a^	↑^a^
Growth	Prey	1	16	540.3	<0.001	26.2	<0.001	↑^a^	↑^a^
Chl *a*/cell	Isolate	1	16	1.58	0.227				
Chl *a*/cell	Light	1	16	6.01	0.025	34.6	<0.001	↓^a^	‐a
Chl *a*/cell	Prey	1	16	17.3	<0.001	36.5	<0.001	↑^a^	‐a
Caro/Chl *a*	Isolate	1	16	6810	<0.001				
Caro/Chl *a*	Light	1	16	2742	<0.001	427	<0.001	↑^a^	↑^a^
Caro/Chl *a*	Prey	1	16	30.2	<0.001	0.70	0.416	↓^a^	↓^a^
Fuco/Chl *a*	Isolate	1	16	363	<0.001				
Fuco/Chl *a*	Light	1	16	157	<0.001	1.58	0.226	↓	↓^a^
Fuco/Chl *a*	Prey	1	16	5.81	0.028	8.94	0.009	↓	‐a
VAZ/Chl *a*	Isolate	1	16	963	<0.001				
VAZ/Chl *a*	Light	1	16	2205	<0.001	96.2	<0.001	↑^a^	↑
VAZ/Chl *a*	Prey	1	16	0.03	0.865	82.7	<0.001	↓^a^	↑

a Significant interaction of prey and light availability (*P *< 0.05).

Changes in pigmentation in response to light and prey availability also differed between isolates (Fig. 4; ANOVA interaction terms, Table 1). Cellular Chl*a* content increased significantly under low‐light compared to high‐light conditions in CCMP1393, but not in CCMP2951 (Fig. 4a; two‐way ANOVAs, Table 1), as already indicated by red fluorescence. Prey depletion was associated with decreases in cellular chlorophyll content in CCMP1393, but there was no significant change in CCMP2951 (Table 1). This pattern is in line with the stronger reduction in FALS and therefore possibly cell size observed in CCMP1393 upon prey depletion (Fig. S2). The adjustment of accessory pigments relative to Chl*a* was more comparable between isolates with fucoxanthin being slightly increased at low‐light intensities (three‐way ANOVA, Table 1). The photo‐protective pigment carotene was present at higher concentrations in CCMP2951 and increased at high‐light intensities in both isolates (three‐way ANOVA, Table 1). The same was true for diadinoxanthin which was present at 13‐fold higher concentrations in CCMP2951 compared to CCMP1393 under prey‐amended conditions when acclimated to near‐optimal high‐light intensity (Figure S3 in the Supporting Information). In low‐light acclimated CCMP1393, diadinoxanthin was not detectable. The de‐epoxidized form diatoxanthin, that is part of the diadinoxanthin cycle in diatoms (Arsalane et al. 1994), was not detected in either *Ochromonas* isolate. The ratio of violaxanthin cycle pigments (VAZ‐pigments) to Chl *a* was comparable among isolates and elevated under high‐light acclimation (Fig. 4d; three‐way ANOVA, Table 1). In CCMP2951, it was significantly higher during prey‐amended compared to prey‐deplete conditions, while the opposite trend was observed in CCMP1393 (two‐way ANOVA, Table 1).

**Figure 5:**
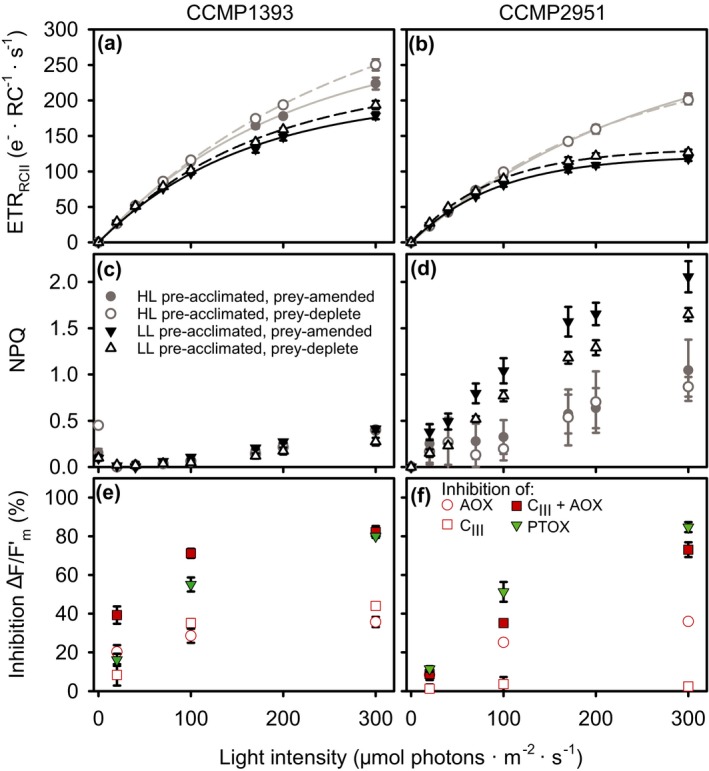
Electron transport in the two *Ochromonas* isolates under prey‐amended and deplete conditions. (a and b) Rapid light–response curves of electron transport through photosystem II. Lines represent photosynthesis–irradiance curves fitted to the data (see Table 2 for parameter values). (c and d) Non‐photochemical quenching (NPQ) in CCMP1393 and CCMP2951 pre‐acclimated to low‐light (15 μmol photons · m^−2^ · s^−1^) and high‐light (100 μmol photons · m^−2^ · s^−1^) intensities under either prey‐amended conditions or after depletion of prey for 3 d. (e and f) Effect of inhibition of mitochondrial respiratory complex III (C_III_), mitochondrial alternative oxidase (AOX), both C_III_ and AOX, or the plastid terminal oxidase (PTOX) involved in chlororespiration on effective photochemical yield of PSII in CCMP1393 and CCMP2951. Measurements were performed with mixotrophic, prey‐amended cultures acclimated to 100 μmol photons · m^−2^ · s^−1^ and exposed to a sub‐saturating light intensity, the acclimation intensity, or a super‐saturating intensity for a period of 3 min prior to measurements. Error bars indicate standard deviation among biological triplicates. [Color figure can be viewed at http://www.wileyonlinelibrary.com]

Maximum ETR_RCII_ (ETR_max_) was reduced in low‐light compared to near‐optimal, high‐light acclimated cultures (Fig. 5, a and b, Table 2) and this effect was strongest in CCMP2951. ETR_RCII_ did not show photoinhibition in response to short‐term exposure to super‐saturating light intensities, even though photoinhibition had been observed in acclimated growth rates of CCMP1393 (Fig. 2a). The capacity for non‐photochemical quenching (NPQ) differed largely between isolates (Fig 5 c and d). CCMP1393 exhibited only modest levels of NPQ, while CCMP2951 showed strong NPQ upon exposure to high‐light intensities, especially in the cultures that had been pre‐acclimated to low‐light intensities. Additionally, for low‐light acclimated CCMP2951, the NPQ response was stronger in prey‐amended compared to prey‐deplete cultures (Holm‐Sidak test, *P *< 0.02 for actinic light >100 μmol photons · m^−2^ · s^−1^).

**Table 2:** Parameters of photosynthesis‐irradiance curve fits to rapid light–response curves of the two *Ochromonas* isolates based on electron transport rates (Fig. 5, a and b). Light intensities are given in μmol photons · m^−2^ · s^−1^. Values in parentheses give estimates of standard errors

Isolate	Light	Prey	ETR_max_	ɑ	adj r^2^
CCMP1393	100	amended	289 (±8)	1.42 (±0.07)	0.998
CCMP1393	100	deplete	370 (±10)	1.37 (±0.05)	0.999
CCMP1393	15	amended	207 (±5)	1.30 (±0.07)	0.996
CCMP1393	15	deplete	232 (±5)	1.35 (±0.05)	0.997
CCMP2951	100	amended	303 (±8)	1.12 (±0.04)	0.999
CCMP2951	100	deplete	271 (±8)	1.19 (±0.06)	0.998
CCMP2951	15	amended	123 (±1)	1.33 (±0.04)	0.997
CCMP2951	15	deplete	133 (±2)	1.52 (±0.06)	0.996

To assess alternative routes of electron transport and their role in photosynthesis, three specific inhibitors were used, targeting PTOX involved in chlororespiration or the mitochondrial electron acceptors C_III_ and AOX, respectively. In both isolates, inhibition of mitochondrial‐ or chloro‐respiration increasingly modified the effective quantum yield of PSII under exposure to increasing light intensities (Fig. 5, e and f; RM‐ANOVA: *F*_2,1_ = 697, *P *< 0.001 for CCMP1393 and F_2,1_ = 491, *P *< 0.001 for CCMP2951). This indicates both pathways act as an overflow mechanism for reducing equivalents produced by the photosynthetic machinery. In CCMP1393, photosynthetic yield was affected by inhibition of C_III_ and AOX both individually and in combination. Inhibition of the entire mitochondrial respiration already affected photosynthetic electron transport at sub‐saturating light intensities (Holm‐Sidak test, *P *< 0.001 for combination of inhibitors compared to each individual one at 20 μmol photons · m^−2^ · s^−1^) and this effect did not increase strongly moving into saturating and supersaturating intensities (100 and 300 μmol photons · m^−2^ · s^−1^, respectively). In CCMP2951, photosynthesis was affected by inhibition of AOX but not by inhibition of C_III_. The simultaneous inhibition of both mitochondrial electron acceptors had no significant effect at sub‐saturating light intensities, but caused a stronger effect compared to inhibition of AOX alone, at higher light intensities (Holm‐Sidak test, *P *< 0.001 for combination of inhibitors compared to each individual one at 100 and 300 μmol photons · m^−2^ · s^−1^).

## Discussion

**Figure 6:**
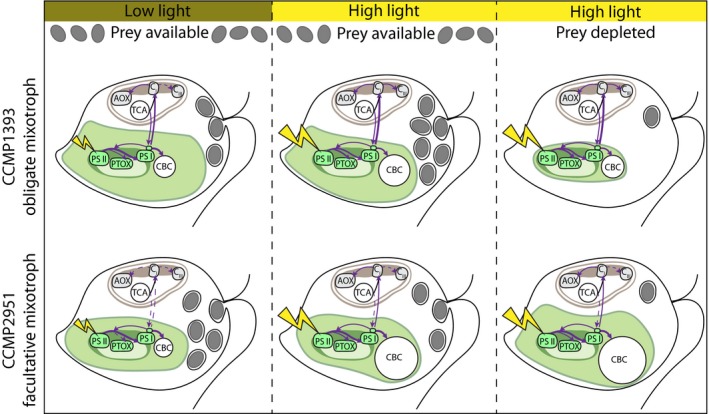
Responses of the two mixotrophic *Ochromonas* isolates to different environmental conditions. Both isolates reach highest growth rates under saturating light and prey availability. The obligate mixotroph (CCMP1393, top row) has higher chlorophyll content under low‐light compared to high‐light conditions and when prey is available compared to prey‐deplete conditions. Rates of both carbon fixation in the Calvin–Benson cycle (CBC) and ingestion of prey are highest under mixotrophic conditions under high light. Reducing equivalents are exchanged between the plastid and mitochondria linking the photosynthetic with the respirational electron transport chain. Growth is most strongly reduced at low‐light intensities. In contrast, the facultative mixotroph (CCMP2951, bottom row) increases its chlorophyll content when prey is depleted at high light, while in the presence of prey light has little impact on chlorophyll content. Rates of ingestion are highest under low‐light condition and maximum rates of carbon fixation are reached when prey is depleted. Export of reducing equivalents from the chloroplast to mitochondria only acts as overflow during high‐light condition. Growth is most strongly reduced in the absence of prey. AOX, alternative oxidase; TCA, tricarboxylic acid cycle; C_I_, respiratory complex I; C_III_, respiratory complex III; PSII, photosystem II; PTOX, plastid terminal oxidase; PSI, photosystem I; CBC, Calvin–Benson cycle. [Color figure can be viewed at http://www.wileyonlinelibrary.com]

The two mixotrophic chrysophytes studied here showed opposite physiological responses to environmental drivers. The capability of CCMP2951 to adjust its nutrition from pure heterotrophy in darkness to mixotrophy in the light and at least survival by autotrophy in the absence of prey characterizes it as a facultative mixotroph, albeit tending more toward the heterotrophic side of the mixotrophic spectrum. Overall, the phenotypic plasticity in its nutrition should facilitate growth under a wide range of environmental conditions. Similar to CCMP2951, CCMP1393 shows very poor autotrophic growth in the absence of prey, although it is able to maintain growth for a few days without feeding (Fig. 2). It might thus have a slightly lower requirement for prey and tend more toward the autotrophic side of the mixotrophic spectrum compared to CCMP2951. CCMP1393 is furthermore unable to grow heterotrophically in darkness, and hence is an obligate mixotroph that requires both light and prey for growth, at least under the conditions tested herein. These different physiologies appear to be rooted in the interaction between photosynthetic and heterotrophic processes, resulting in distinct ecological strategies for these two chrysophytes. The two isolates thus likely represent different species, as also supported by morphological differences with an eyespot present in only CCMP2951 and detectable differences in their 18S rRNA genes. While their 97% nucleotide identity in 18S rRNA genes indicates close relatedness, this gene is strongly conserved and does not always provide sufficient resolution to distinguish closely related species. The percentage of shared genes in their genomes might, for instance, be much lower as reported in two green algal *Micromonas* species (Worden et al. 2009). Overall, our results exemplify the difficulties in linking phylogenetic to functional diversity of mixotrophs (Figs. 1 and 6).

Organisms with a mixotrophic lifestyle face a trade‐off between investments into nutrient uptake, photosynthesis, and phagotrophy (Andersen et al. 2015). Modeling studies suggest that differential investment into optimization of these traits underlies successional patterns of phytoplankton (Berge et al. 2017). Similarly, the high phenotypic plasticity in the facultative mixotroph CCMP2951 observed here reflects differential investment into these traits that is shaped by environmental conditions. At low‐light intensities, increased ingestion rates substitute for photosynthesis and make additional investment into chlorophyll biosynthesis obsolete. The capacity of phagotrophy to sustain growth in darkness is in agreement with previous studies of predominantly heterotrophic freshwater isolates of *Ochromonas* and *Poterioochromonas* (Andersson et al. 1989, Sanders et al. 1990, Wilken et al. 2013), although its maximum growth and ingestion rates observed here are lower than reported for freshwater *Ochromonas* isolates (Rothhaupt 1996, Wilken et al. 2013). Moreover, the high nutritional plasticity requires that the two nutritional pathways can principally function independently from each other, as both can supply sufficient resources to fuel cellular metabolism. In particular, under mixotrophic growth at low‐light compared to high‐light intensities, higher rates of ingestion seem to compensate for the decreased rates of carbon fixation resulting in an inverse relationship between these two rates (Fig. 3). Thus, our results demonstrate that photosynthesis and heterotrophy act as partly substitutable routes of resource acquisition in the facultative mixotroph.

Unlike facultative mixotrophy, photosynthesis and heterotrophy covary in the obligate mixotroph CCMP1393 (Fig. 3) and thus constitute complimentary routes of resource acquisition that cannot function independently from each other. Such a strategy cannot be explained solely by differential investment into resource acquisition traits. Instead, the reliance on the simultaneous performance of both photosynthesis and phagotrophy to attain positive growth rates suggests an integration of these two nutritional pathways that restrict their independent execution. This interdependence of photosynthesis and heterotrophy results in decreased ingestion rates under light limitation and decreased chlorophyll content when prey are lacking, in line with changes in transcriptional patterns observed in this isolate (Lie et al. 2018). Dependency on light for survival has been described in other mixotrophs such as the brackish chrysophyte *Ochromonas minima* (Flöder et al. 2006) and several dinoflagellates (Jeong et al. 2010, Hansen 2011). The increased chlorophyll content in CCMP1393 under low‐light conditions (Figs. 2a and 4a) reflects a photoacclimation response typical for photoautotrophs that results in greater light‐harvesting capacity under low light to help maintain photosynthesis (MacIntyre et al. 2002). Despite the potential for photosynthetic carbon acquisition, this route is not sufficient to support stable positive growth rates in CCMP1393 under the conditions tested herein (Fig. 2c). A dependence on phagotrophy is usually explained as reflecting a mechanism for acquiring essential growth factors or nutrients from prey. For example, increasing rates of phagotrophy have been observed with increasing light intensity in the freshwater chrysophyte *Dinobryon cylindricum*, and attributed to providing necessary nutrients via prey cell digestion (Caron et al. 1993). Similar dependencies were reported in the chrysophyte *Uroglena americana*, which appears to be more closely related to CCMP1393 than CCMP2951, although support in this region of the tree is largely lacking (Fig. 1), and the dependency was further characterized as being a requirement for bacterially produced phospholipids (Kimura and Ishida 1989). A recent study on CCMP1393 did not detect transcripts that encode nitrate transporters, and concluded that this chrysophyte does not have the capacity to utilize nitrate (Lie et al. 2018). Additionally, based on growth experiments the freshwater chrysophyte *Ochromonas globosa* relies on reduced nitrogen sources (Wilken et al. 2014b). However, in our experiments, both ammonium and nitrate were provided which would presumably obviate reliance on prey as nitrogen source depending on ammonium depletion rates, which can be rapid in algal culture experiments (McDonald et al. 2010). In general, biosynthetic pathways required for purely photoautotrophic growth could have been lost by some constitutive, obligate mixotrophs, although in the absence of complete genome sequences the prevalence of such pathway reductions remains an open question.

An obligate requirement for phagotrophy in photosynthetic eukaryotes can be expected to result in a finely tuned integration of photosynthetic and heterotrophic metabolisms, regardless of whether this is the cause or consequence of an obligately mixotrophic lifestyle. A close energetic coupling between plastids and mitochondria involving an overflow mechanism for excess reducing equivalents, as well as support of carbon fixation by mitochondrially derived ATP, also occurs in diatoms (Allen et al. 2008, Bailleul et al. 2015). Effects of mitochondrial inhibitors must generally be interpreted with care, as indirect effects are difficult to exclude. Nevertheless, in CCMP1393, photosynthetic electron transport seems to be strongly influenced by mitochondrial respiration (Fig. 6). In addition to its role as an overflow mechanism for reducing equivalents observed in both isolates, mitochondrial respiration acts as an electron sink even under sub‐saturating light conditions in CCMP1393. It therefore likely represents an integral part of photosynthetic electron flow in this obligate mixotroph, involving both AOX and C_III_, which might be used for ATP production from photosynthetically derived electrons. While this could potentially reduce the need for heterotrophic respiration and hence increase carbon assimilation efficiencies from prey, higher rates of carbon fixation in the presence of prey do not indicate a tendency toward photoheterotrophy as suggested for the freshwater chrysophyte *Ochromonas danica* (Wilken et al. 2014a). In the facultative mixotroph, CCMP2951 mitochondrial respiration was negligible as an electron sink under low‐light intensities and the overflow mechanism at higher light intensities mainly relied on alternative oxidase, which does not contribute to the proton gradient for ATP production (Vanlerberghe and McIntosh 1997). Hence, in contrast to the obligate mixotroph, mitochondrial respiration is not required as an integral part of photosynthetic electron transport in the facultative mixotroph.

Differences in phenotypic plasticity between the two isolates studied here are further reflected in their responses to super‐saturating irradiances. In the obligate mixotroph, growth rates were reduced under long‐term exposure to high light despite the maintenance of high electron transport rates during short‐term exposure. This indicates a downstream limitation to electron transport that could not be counteracted by photoacclimation. The lower content of the photoprotective pigments carotene, and diadinoxanthin in the obligate mixotroph, relative to the facultative mixotroph, might contribute to its higher susceptibility to photoinhibition. The light‐induced de‐epoxidized form of diadinoxanthin, diatoxanthin, is responsible for some forms of NPQ in other stramenopiles, specifically diatoms (Arsalane et al. 1994). However, we did not detect this pigment in either *Ochromonas* isolate, consistent with its absence in other chrysophytes (Lichtlé et al. 1995, Tanabe et al. 2011). Differences in VAZ pool size might underlie the higher NPQ capacity in CCMP2951, but cannot fully explain the large difference between isolates. These might be due to other components involved in NPQ such as the ancient light harvesting related protein (LHCSR and LHX) found in diatoms and green algae (Peers et al. 2009, Bailleul et al. 2010, Guo et al. 2018). In addition, the formation of a pH gradient across the thylakoid membrane is required for the induction of NPQ. In CCMP1393, a slight NPQ response is already present in darkness, indicative of a pH gradient formed through chlororespiration (Jakob et al. 1999). If the close interaction between plastids and mitochondria in this isolate supports a pH gradient across the thylakoid membrane independently of light, a strong NPQ response might not be beneficial as it could restrict photosynthesis at low‐light intensities.

The two mixotrophic strategies described here likely reflect adaptation to different niches, although the specific competitive advantages gained within complex communities is difficult to assess and the environmental distributions of mixotrophic strategies remain largely unstudied. A first assessment of the biogeography of mixotrophs revealed a gap in information regarding non‐dinoflagellate constitutive mixotrophs (Leles et al. 2019), such as the chrysophytes studied here. In modeling studies, constitutive mixotrophs are typically considered to be primarily autotrophic, supplementing their nutrition by ingestion of prey if either light or nutrients become limiting and thus have an advantage under nutrient limitation (Leles et al. 2018). This is a strategy, for example, found in prymnesiophytes and some dinoflagellates (Hansen and Hjorth 2002, Hansen 2011). However, the spectrum of constitutive mixotrophs, ranging from preferentially heterotrophic to obligately mixotrophic is less commonly addressed. In a similar manner to which primarily autotrophic mixotrophs have an advantage competing for nutrients against non‐phagocytotic photoautotrophs in nutrient limiting conditions, primarily heterotrophic mixotrophs could have an advantage over purely heterotrophic taxa at low prey availability, if sufficient light is available (Tittel et al. 2003, Fischer et al. 2017). Both types of mixotrophic strategies could thus be advantageous in oligotrophic environments. Obligate mixotrophy has been implemented in modeling studies in the form of kleptoplasty, where the capacity of a heterotrophic protist to photosynthesize depends on its ingestion of autotrophic prey (Mitra et al. 2016). Although based on different underlying physiology, kleptoplastic protists dominated predicted plankton biomass in a nutrient‐limited scenario (Leles et al. 2018), hinting at the success of obligate mixotrophs in oligotrophic waters. From our results, the narrow range of environmental conditions that permit growth of the obligate mixotroph might seem disadvantageous, but this strategy is likely efficient in stable environments with low‐resource availability. Additionally, while the facultative mixotroph still reached a higher percentage of its maximum growth rate under light limitation, this advantage might disappear under low but stable availability of light and prey, when close integration of photosynthesis and heterotrophy in the obligate mixotroph presumably preserves resources. Moreover, export of photosynthetically derived electrons to the mitochondrial respiratory chain, even under low‐light conditions, could reduce the need to respire ingested carbon and therefore allow higher carbon assimilation efficiencies. Obligate mixotrophy could hence represent a specialization for stable resource‐limited environments such as the oligotrophic oceans, while facultative mixotrophy would be more advantageous under fluctuating conditions as found in shallower coastal regions.

The opposite responses to environmental drivers observed in the two marine *Ochromonas* species studied here suggest that even closely related mixotrophic protists will respond differently to changing ocean conditions with differing consequences for carbon cycling through marine microbial food webs. For instance, the nutritional balance of mixotrophs might directly be affected by warming with a shift toward a more heterotrophic nutrition at higher temperatures (Wilken et al. 2013, Worden et al. 2015). Such a shift would be more likely in facultative mixotrophs that have greater nutritional flexibility than in obligate mixotrophs which have a close interdependency of photosynthesis and heterotrophy. Further, expansion of oligotrophic regions as oceans warm (Polovina et al. 2008) may provide conditions favoring the resource efficient strategy, i.e., obligate mixotrophy. Understanding the physiological basis of different mixotrophic strategies in a broader assortment of environmentally relevant models will help to predict their respective biogeography and contributions to food web dynamics and carbon cycling in the future oceans.

## Author contributions

AZW and SW planned and designed the research; SW performed experiments and analyzed physiology data; and CJC performed sequencing and phylogenetic analysis. SW drafted the manuscript with input from AZW and additional edits from CJC.

## Supporting information


**Figure S1.** Abundances of *Ochromonas* and the bacterial prey *Vibrio fischeri* in semi‐continuous cultures of the two *Ochromonas* isolates with or without daily prey‐amendments.

**Figure S2.** Forward‐angle light scatter (FALS) of *Ochromonas* isolates under prey‐amended and prey‐deplete conditions.

**Figure S3.** Molar ratio of diadinoxanthin content relative to chlorophyll *a* in the two *Ochromonas* isolates under prey‐amended and prey‐deplete conditions.
