# Supplementary figures and images for: Contrasting Mixotrophic Lifestyles Reveal Different Ecological Niches in Two Closely Related Marine Protists
Source: J Phycol. 2019 Nov 1;56(1):52–67. doi: 10.1111/jpy.12920 (PMC7065223; doi:10.1111/jpy.12920)

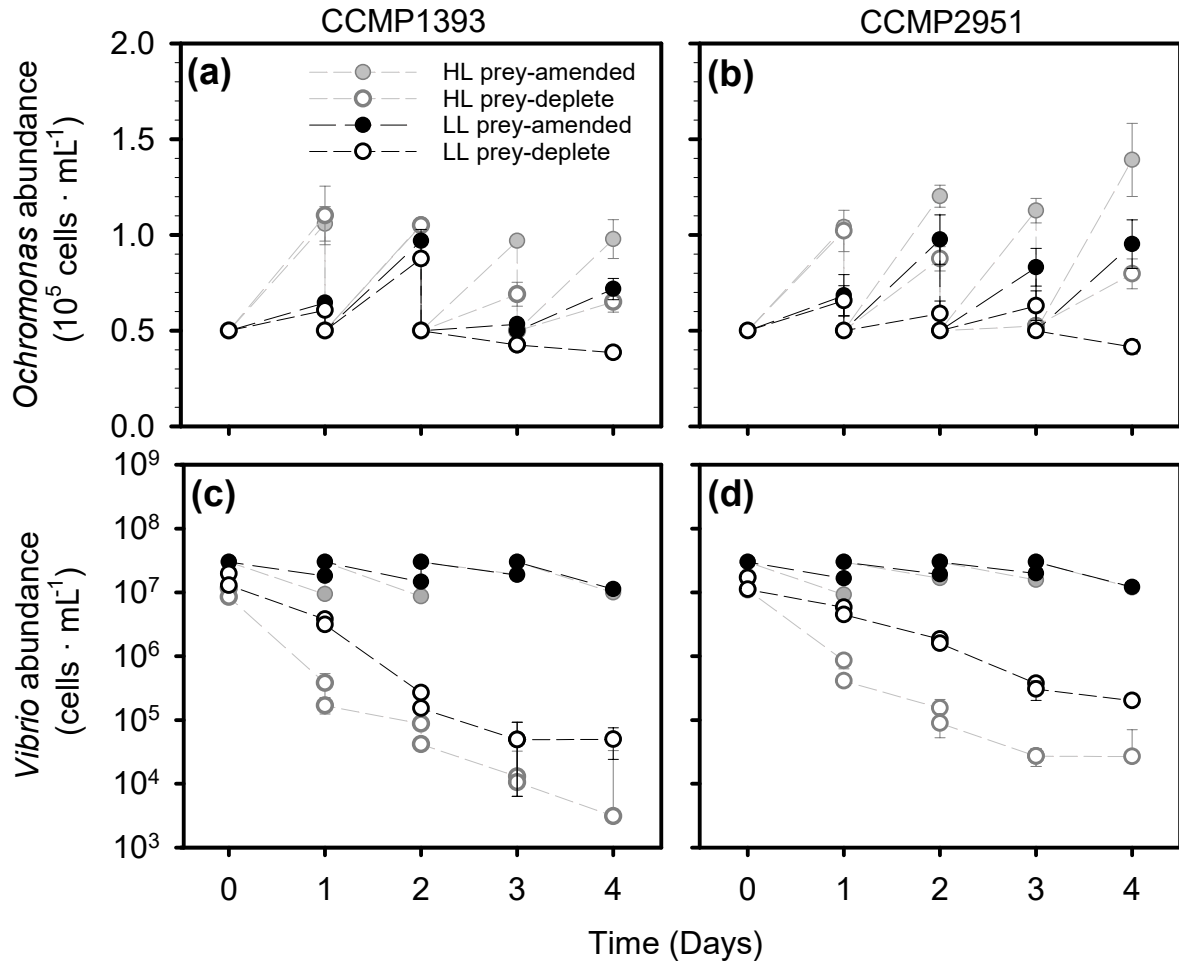

Supplement: Supplementary file 1 — Figure S1. Abundances of Ochromonas and the bacterial prey Vibrio fischeri in semi‐continuous cultures of the two Ochromonas isolates with or without daily prey‐amendments. [file JPY-56-52-s001.pdf]

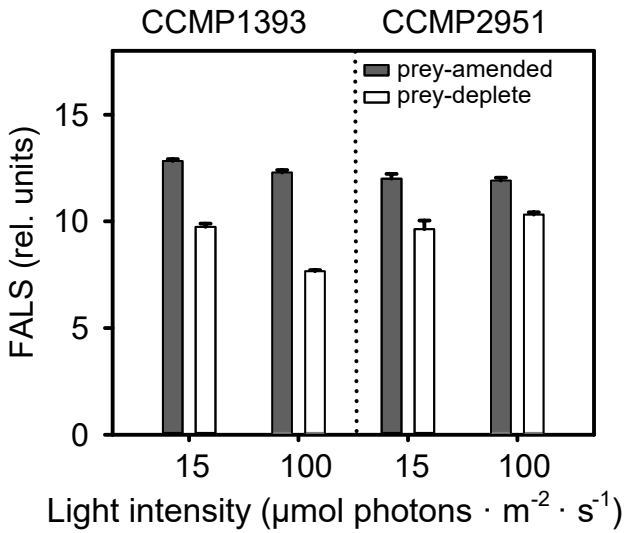

Supplement: Supplementary file 2 — Figure S2. Forward‐angle light scatter (FALS) of Ochromonas isolates under prey‐amended and prey‐deplete conditions. [file JPY-56-52-s002.pdf]

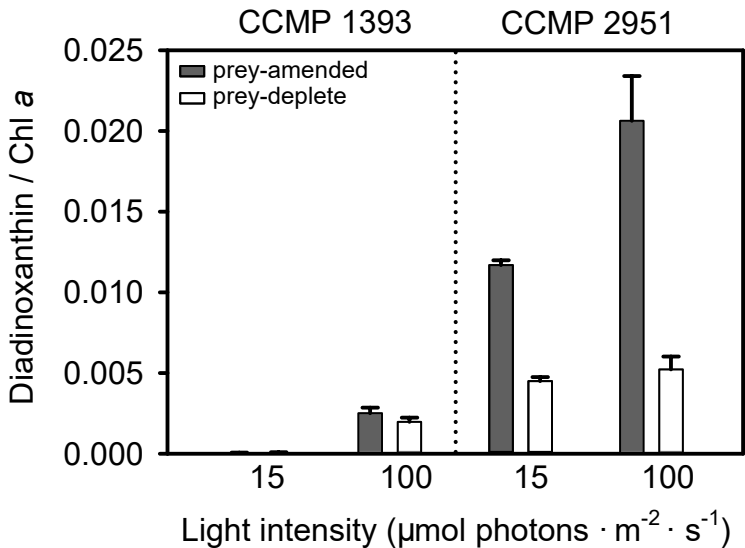

Supplement: Supplementary file 3 — Figure S3. Molar ratio of diadinoxanthin content relative to chlorophyll a in the two Ochromonas isolates under prey‐amended and prey‐deplete conditions. [file JPY-56-52-s003.pdf]
